# Increasing health service access by expanding disease coverage and adding patient navigation: challenges for patient satisfaction

**DOI:** 10.1186/s12913-020-5009-x

**Published:** 2020-03-06

**Authors:** Russell K. Schutt, Mary Lou Woodford

**Affiliations:** 1 Department of Sociology, University of Massachusetts Boston and Harvard Medical School, Boston, MA 02125-3393 USA; 2 MLW Associates, LLC, Southborough, USA

**Keywords:** Women’s health, Cancer screening satisfaction, Health disparities, Case management, Patient navigation

## Abstract

**Background:**

Cancer control programs have added patient navigation to improve effectiveness in underserved populations, but research has yielded mixed results about their impact on patient satisfaction. This study focuses on three related research questions in a U.S. state cancer screening program before and after a redesign that added patient navigators and services for chronic illness: Did patient diversity increase; Did satisfaction levels improve; Did socioeconomic characteristics or perceived barriers explain improved satisfaction.

**Methods:**

Representative statewide patient samples were surveyed by phone both before and after the program design. Measures included satisfaction with overall health care and specific services, as well as experience of eleven barriers to accessing health care and self-reported health and sociodemographic characteristics. Multiple regression analysis is used to identify independent effects.

**Results:**

After the program redesign, the percentage of Hispanic and African American patients increased by more than 200% and satisfaction with overall health care quality rose significantly, but satisfaction with the program and with primary program staff declined. Sociodemographic characteristics explained the apparent program effects on overall satisfaction, but perceived barriers did not. Further analysis indicates that patients being seen for cancer risk were more satisfied if they had a patient navigator.

**Conclusions:**

Health care access can be improved and patient diversity increased in public health programs by adding patient navigators and delivering more holistic care. Effects on patient satisfaction vary with patient health needs, with those being seen for chronic illness likely to be less satisfied with their health care than those being seen for cancer risk. It is important to use appropriate comparison groups when evaluating the effect of program changes on patient satisfaction and to consider establishing appropriate satisfaction benchmarks for patients being seen for chronic illness.

## Background

Patient satisfaction is an important goal for health care services, as both an indicator of service quality and as an influence on subsequent service utilization [1–5]. Increasing patient satisfaction with care delivery may thus provide a means for improving engagement in and outcomes of care delivery [6] and was designated by the American Cancer Society’s Patient Navigation Leadership Summit as one of the key metrics for evaluating patient navigation programs [7].

In spite of some positive evidence, however, and the clear potential for patient navigation programs to improve care delivery to underserved populations, research has not consistently linked patient navigation programs to increased satisfaction [8–11]. A systematic review of satisfaction outcomes from nine patient navigation studies that had a comparison group reported that three studies found positive effects of patient navigation on overall patient satisfaction, but in a pooled meta-analysis of data from five studies, only one found that patient navigation increased patient satisfaction with cancer care (although that study was the only one of the five that was a randomized controlled trial) [12]. The multisite Patient Navigation Research Program (PNRP) found that patient navigation improved satisfaction only among socially disadvantaged patients at two sites serving patients with recent breast or colorectal cancer diagnoses [13, 14].

One reason for inconsistent effects of patient navigation on satisfaction with care may be that studies have not taken into account the financial, practical, and cultural barriers that patients may confront and the ability of patient navigators to overcome them [15–17]. Reducing some barriers may increase patient satisfaction, but this may not occur with all barriers [11]. In addition, more complex health care problems that are more difficult to treat may be less likely to have satisfying outcomes [18]. Some of the variation in prior research findings may also reflect differences in influences on satisfaction with health care overall compared to satisfaction with specific health care experiences [19, 20].

Sociodemographic characteristics may also influence patient satisfaction and so must be taken into account. Prior research identifies age as a consistent predictor of greater satisfaction, in spite of its association with declining health [19, 21, 22]. Satisfaction also has been associated in prior research with better self-reported health [21, 23], fewer symptoms of depression [23, 24], and English proficiency [25]. African Americans and immigrants have faced particular barriers to health care that have resulted in lower levels of satisfaction [26–28]. In addition, higher levels of education have been associated consistently with lower levels of satisfaction [20, 21].

The broader research literature on patient satisfaction thus suggests that inconsistent findings about the effect of patient navigation programs on patient satisfaction may be due in part to insufficient controls for other influences that may explain or alter these effects. The addition of patient navigators to an established cancer detection program created an opportunity to test these possibilities.

### The program redesign

The National Breast and Cervical Cancer Early Detection Program (NBCCEDP) initiated by Congress in 1990—and the case management component added in 1998—improved access to breast and cervical cancer screening for low income, underserved women by funding programs in every state, territory, and tribe in the United States [29, 30]. In many states, NBCCEDP nurse case managers followed up with women identified as at high risk for breast or cervical cancer and encouraged them to seek conclusive diagnostic testing and, when warranted, referral to treatment. However, outcome evaluations revealed that screening disparities related to socioeconomic status remained [31–33].

After a state-wide program evaluation led by the authors identified lower rates of screening and referral among eligible women with immigrant backgrounds, an Expert Panel recommended two major program changes: (1) Community-based patient navigators were added to improve outreach at each health care delivery site; (2) The patient navigators were also expected to assist with management of the chronic illnesses that both increased cancer risk and created barriers to screening and treatment. The CDC granted a waiver of the original program requirements to allow the inclusion of chronic illness as well as cancer risk; financial eligibility continued to be limited to under- and uninsured persons at or below 250% of the federal poverty level (corresponding to $27,925 for one person in 2012 in U.S. dollars).

The case managers at each site continued to focus on diagnostic screening and referral to treatment for patients at high risk of breast or cervical cancer, while the patient navigators worked with program patients to improve their management of such chronic diseases as hypertension, asthma, and diabetes. The patient navigators were hired from the most underserved communities and so two-thirds were Hispanic and fully nine in ten were bilingual. Although the patient navigators were not expected to have any formal medical education, a 50-h patient navigation training program was designed for them and delivered at a central location. A program director supervised both patient navigators and case managers at each service delivery site.

### Study aims

This study examined multiple satisfaction measures in the program before and after the redesign. Patient characteristics were controlled as possible confounders, perceived barriers were examined as potential mediators, and four measures of different aspects of satisfaction were included as outcomes. Specifically, the analysis is designed to answer three interrelated research questions: (1) Did patient diversity increase after the program redesign? (2) Did any aspects of health care satisfaction increase among those from ethnic or immigrant backgrounds, as intended by the program redesign? (3) Did sociodemographic characteristics or perceived barriers explain improvements in satisfaction levels?

## Methods

Surveys of representative samples of program patients conducted by the University’s Center for Survey Research before and after the program change created a repeated cross-sectional design (“trend study”) [34] that allowed before-after comparisons of patient characteristics, perceived barriers, and average satisfaction levels.

### Study participants

Both samples were selected randomly from the public health agency’s database of program service recipients, allowing study eligibility to be determined prior to patient contact. The eligible population for the “pre” survey in 2005 consisted of the 3178 women who had received any case management services from the program in the preceding 2 years (2003–2004) at any of its 26 sites, had been screened for breast or cervical cancer, and spoke English, Spanish, or Portuguese. Of 385 patients sampled randomly for interviews, 76% (293) could be located and 71% of these were interviewed, for a final sample size of 207 (54% of the initial sample). The eligible population for the “post” survey in 2012 consisted of the patient population with either case management or patient navigation services in the preceding year. By that time, program enrollment had expanded as a result of the program changes to 10,627 (but with the number of program delivery sites statewide reduced to 17). Of 427 patients randomly selected from those who met eligibility criteria (could be located and had received health care at the selected site in the preceding year and could speak English, Spanish, or Portuguese), 383 (90%) completed interviews (33% of the initial random sample prior to checking for eligibility).

All procedures were approved by the University’s Institutional Review Board for the Protection of Human Subjects. Enrolled patients received a letter prior to the interviews informing them that participation in the phone survey was completely voluntary and confidential and would not affect their program services. These points were repeated in the introduction on the phone and the interview did not proceed unless patients consented verbally.

Questionnaires were translated into Spanish and Portuguese, using forward and back translation and then reconciling differences; in both surveys, interviews were conducted in English, Spanish, or Portuguese based on patient preference (see supplementary files 1 and 2). There were two differences in selection of patients in the before and after surveys. (1) Prior to the program redesign, women only received active case management when follow-up testing arranged by the case manager yielded an abnormal finding indicating high risk of breast or cervical cancer. Since the focus of this first survey was on experience with case managers, the sample was stratified by risk and high risk cases (335) were then sampled disproportionately (50 in the intended sample were in the low risk group). Response rates were similar for both the high and low risk groups and after weighting to adjust for the disproportionate selection of high risk cases, the obtained sample was similar in ethnicity and primary language to the program population, but somewhat more educated (89% of the obtained sample had completed high school, compared to 72% of the program population). Only respondents who knew of the program and answered questions about their satisfaction with it are included in the comparative analysis (157 of 207).

(2) After expanded eligibility criteria following the program change, many more men were eligible for program participation and comprised 16% (*n* = 61) of the “post” sample, but since men were not included in the first survey they were omitted from this analysis (resulting in a sample size of 320). Although survey respondents could have differed in multiple ways from the program population, there were no statistically significant demographic and location differences in comparison to the total program population other than underrepresentation of patients at two sites and overrepresentation at one.

### Measures

Health care satisfaction was operationalized with four indicators that ranged in their focus from more general to more specific (see Table 1 for details) [6, 23]: (1) satisfaction with the program overall; (2) satisfaction with the program (first survey) or with the specific health clinic where program services had been received (second survey, because program services were then less distinct within the clinics); (3) satisfaction with the program staff with whom the respondent had had contact. The measures of satisfaction with program staff were then used to construct two measures of change: satisfaction with the licensed case manager at both times for those who had a case manager in the revised program and satisfaction with the licensed case manager in the initial program and with the patient navigator in the revised program for all respondents [35, 36].

**Table 1:** Measures

Concept/Variable	Question(s)	α	Notes
*Satisfaction*			
Overall satisfaction with health care	How would you rate the overall quality of the health care that you have received in the past year		In the past year. On a scale from 0 to 10, where 0 is the worst health care possible and 10 is the best health care possible
Program/clinic satisfaction	Would recommend to a friend; location convenient, satisfied overall, doing better because of it	.85	
Staff satisfaction	CM (2005); PN (2012)	.80	
CM satisfaction	How satisfied were you with the manner in which the **case manager…**kept in touch, helped find resources, helped communicate, helped with transportation, helped scheduling	.76	Responses: very satisfied, satisfied, dissatisfied, very dissatisfied
*Barriers*	How much of a problem has ___ been for you in getting **health care** that you required?		Responses: major problem, sometimes a problem, minor problem, no problem at all
Economic	Bills, insurance, distrust system	.71	
Fear	Bad news, pain	.71	
Practical	Transportation, system knowledge, scheduling appointments, time for tests and treatment	.62	
Immigration	Your citizen or immigration status		Non-immigrants coded as having “no problem at all.”
*Health care access*	Aids to access		Cost didn’t prevent medical care; Regular place for healthcare; One person as provider, Have insurance source
Physical health	Would you say that in general your health is …?		Responses: excellent, very good, good, fair or poor
Distress	Bothered in last 2 weeks by …feeling down, …little interest in doing things	.76	Responses: not at all, several days, more than half the days, nearly every day
Preferred language	Choice of English, Spanish, or Portuguese for interview		2012 survey questions indicated respondents interviewed in Spanish or Portuguese were first generation
*Sociodemographics*			
Race/ethnicity	Are you of Spanish, Hispanic or Portuguese descent?; What race do you consider yourself?		
Age	In what year born		
Education	Highest year of education		
*Program status*			
Cancer risk	Any cancer concerns in past year?		
Patient navigator contact	Recognize PN name		Reported by 21% of the post-program patients interviewed
Case manager	Had CM (records)		

Indexes corresponding to three types of barriers to health care were identified with responses to a set of questions about “How much of a problem has ___ been for you in getting **health care** that you required?” [37–39]. An exploratory factor analysis of patient responses about 11 specific barriers in both samples identified three factors, which are represented with indexes constructed as simple averages [40]: economic, fear, practical. One question about immigration status as a barrier was asked only of those who were immigrants.

Health care access was indicated with questions about sources of health care insurance and ability to afford needed medical care [41, 42]. Physical health status was measured with the standard self-report rating [43, 44], while mental health was operationalized with a two-question index of depressive symptoms [45]). Age and education were obtained from program records. Ethnic and racial identification were identified with standard questions. Preference for speaking Spanish or Portuguese (in the interview) was used to distinguish first generation status, as it did in the second survey when a specific question was added about immigration status [26, 46].

Three indicators of program status that would have been affected by the program are distinguished separately to clarify the role of the program change itself. Cancer risk—which would have been less common after the program due to the expanded enrollment criteria—was identified with one question. Recognition of the patient navigator’s name in 2012 was indicated by a dichotomous indicator and was used to control for the extent of contact. All respondents had a case manager in 2005 and a patient navigator in 2012, but those being seen only for chronic illness in 2012 did not have a case manager, so having a case manager in 2012 in addition to a patient navigator was distinguished with a separate dichotomous indicator.

### Statistical analyses

Bivariate tests (t-tests and χ^2^ tests) were used to identify changes in the composition of the program’s patients, including in race and ethnicity, marital and employment status, and health and health care access. U.S. Census data for the state were used to indicate trends in the state population that might explain changes in ethnic diversity within the program. T-tests were also used to identify differences in levels of each of the satisfaction indicators before and after the program change, and to compare satisfaction with the case manager to satisfaction with the patient navigator for patients who had both after the program change.

Multiple regression analysis (OLS) was used to determine the independence of any pre-post program change differences in satisfaction levels from program status indicators, and the patient characteristics and access barriers identified in the literature as potential predictors. Tests of interaction effects (using product terms) indicated whether differences in satisfaction levels after the program change occurred only for the ethnic and racial groups that had previously been less satisfied [47].

Standardized coefficients (β) are reported for the regression analyses first using only the program status indicators (Model 1) and then including all potential influences on satisfaction (Model 2). The coefficient of determination (R^2^) was used to assess and compare the fit of each model, while R^2^ Δ was used to identify the contribution of each block of factors potentially associated with satisfaction. Separate multiple regression analyses were conducted without the terms representing the interaction of race/ethnicity and program, but since the coefficients for the other terms in these models changed little after the interaction terms were added, only the final models including both main effects and interaction terms are presented (in Model 2).

## Results

After the program redesign, enrollment data showed that patients were older, less educated, more likely to be Hispanic or African American and to have been interviewed in Spanish or Portuguese, and more likely to be living alone and to not be working (see Table 2). The proportion of the program’s patients who were Hispanic and African American increased dramatically, by 206 and 295%, respectively.

**Table 2:** Characteristics of Program Patients Before and After Program Redesign

	Before	After	χ^2^ (or t), p
Age (Years)	40.1	53.0	t = −12.71^***^
Education (<High School Grad)	8.2%	28.3%	23.56^***^
Hispanic	14.6%	44.7%	41.88^***^
African American	3.8%	15.0%	13.11^***^
Spanish interview	6.4%	32.5%	39.54^***^
Portuguese interview	0.6%	9.4%	13.23^***^
Ever Married	30.1%	35.0%	1.13
Living Alone	22.9%	41.3%	13.47^***^
Working	67.7%	51.6%	11.14^***^
Have Health Insurance	61.1%	95.0%	89.17^***^
Regular Place for Care	93.6%	99.1%	11.82^***^
Cost Did Not Deter Care	49.7%	79.5%	44.13^***^
Poor Self-Rated Health	2.58	3.07	5.27^***^
Any Physical Health Problems	60.3%	89.4%	55.23^***^
Symptoms of Depression	.53	.83	t = 3.51^***^
Knows of Cancer Diagnosis	27.4%	13.5%	14.79***
N (aprx., varies)	157	378	

*** *p < =.001*

Access to health care had also improved dramatically by the second survey (Table 2). Patients in the redesigned program were much more likely to report having health insurance (with almost universal coverage), to report having a regular place and a regular doctor for health care, and not to have been deterred from getting needed medical care by its cost. However, as expected after the shift in emphasis to chronic illness, levels of self-reported overall health, physical health problems, and symptoms of depression each indicated poorer average health in the post-program change sample.

Patient satisfaction was higher with the quality of health care overall after the program redesign, but lower with respect to the clinic from which they received program services and lower with respect to the patient navigator after the change compared to the case manager prior to the change (see Table 3). However, there was no difference in average satisfaction with the program case manager between those in the original program and the subset of respondents in the redesigned program who had a case manager as well as a patient navigator.

**Table 3:** Satisfaction Indicators by Program^a^

Satisfaction with…	Pre μ (N)	Post μ (N)	T	*p* < =
Care Quality	8.20 (157)	8.78 (320)	−3.52	.001
Program or Clinic	3.67 (157)	3.38 (320)	6.24	.001
Staff (CM or PN)	3.46 (157)	3.34 (283)	1.95	.05
Case Manager (CM)	3.46 (157)	3.45 (46)	.23	NS

^a^Women only

By contrast, satisfaction with the patient navigator was higher than satisfaction with the case manager for patients who had both a patient navigator and a case manager after the program change (in other words, for those being seen for cancer risk rather than only for chronic illness) (t = 2.01, df = .47, *p* ≤ .05).

The multiple regression analyses of satisfaction in relation to program status identified the same apparent effects of the program change (Model 1 in Table 4), but suggested different bases for these effects with the different satisfaction indicators (Model 2 in Table 4). Controlling for patient background entirely explained the improved satisfaction with health care overall in the redesigned program. However, the controls did not explain the decline in satisfaction with the program or clinic or with the care provider (CM/PN) between respondents in the redesigned program and those in the original program. Patients who had a case manager in addition to a patient navigator were more satisfied with the program/clinic and with program staff than those who only had a patient navigator (although the effect on program/clinic satisfaction only emerged after other variables were controlled), and this difference was not explained by the controls (Model 2 in Table 4). There was no statistically significant difference related to the program status indicators in satisfaction with the program case manager for the subset who also had a case manager in the redesigned program.

**Table 4:** Regression Analysis of Satisfaction Indicators^a^

*Measure*	*Health Care Satisfaction*		*Program/Clinic Satisfaction*^*b*^		*CM/PN Satisfaction*^*b*^		*CM Satisfaction*^*b*^	
*Model*	*1*	*2*	*1*	*2*	*1*	*2*	*1*	*2*
*Program Status (R*^*2*^*Δ)*		*.01*		*.05*		*.04*		*.01*
CC Program (Post)	.14^**^	−.04	−.30^***^	−.31^***^	−.14^**^	−.24^***^	−.02	.04
Knows of cancer	−.05	.07	−.01	−.02	−.03	−.01	−.02	−.04
Had CC case manager	.06	.05	.07	.10^*^	.11^*^	.15^***^	–	–
Recognized navigator	−.02	.05	.04	.05	.03	.04	.01	−.12
*Patient (R*^*2*^*Δ)*		*.05*		*.07*		*.06*		*.15*
Hispanic		.04		−.15		−.01		.10
Spanish interview		.22**		.08		.06		−.08
Portuguese interview		.18**		.17^**^		.03		.03
African American		.10		.07		.09		−.01
Education		−.03		.11^*^		.05		.10
Age		.11*		.08		.24^***^		.36^***^
Not working		.01		.02		−.05		−.20^**^
Poor self-rated health		−.07		−.11^*^		−.10		−.09
Depression symptoms		.01		.01		−.05		−.04
Cost deterred care		−.02		−.11^*^		−.08		−.04
*Barriers*^*b*^*(R*^*2*^*Δ)*		*.09*		*.05*		*.08*		*.08*
Economic		.10		.15^**^		.10		.18^*^
Practical		.26^***^		.15^**^		.25^***^		.16^*^
Fear		.02		−.07		−.07		.02
Immigration status		.00		−.04		−.01		.05
*Interactions (R*^*2*^*Δ)*		*.03*		*.02*		*.01*		*.00*
Hispanic*CC		−.09		.06		.05		.01
Spanish*CC		−.07		.07		−.05		.07
Portuguese*CC		−.01		−.03		.02		−.00
Black * CC		−.18^***^		−.10		−.10		−.04
R^2^	.03	.20	.08	.21	.02	.18	.00	.26
N	448	448	448	448	415	415	187	187

^a^Standardized beta

^b^Satisfaction measures scored so higher scores mean more satisfaction; barriers scored so that higher scores mean lower perceived barriers

* *p < =.05;*

**p < =.01;

*** *p < =.001*

The independent effects of patient characteristics and barriers also differed between the four satisfaction indicators. Patients who were interviewed in Spanish or Portuguese were more satisfied with their health care overall, as were those who were older. Satisfaction with the program or clinic was also higher among Portuguese speakers, and associated positively with education, self-rated health, and lack of a cost deterrent, but unrelated to age. Staff satisfaction and case manager satisfaction were both positively associated with age, while case manager satisfaction was lower among those who were not working. All four satisfaction indicators were associated with more perceived practical barriers to health care, while program/clinic satisfaction and case manager satisfaction were also diminished by more perceived economic barriers. Perceived barriers due to fear and immigration status were not associated with satisfaction. Among post-change patients only, African American patients were less satisfied with health care overall than were others, but there were no other statistically significant interactions with ethnicity or language. The regression analyses explained about one-fifth of the proportion of variance in each satisfaction indicator (a bit more for the smaller sample available for the regression analysis of satisfaction with the case manager).

## Discussion

After the redesign, as intended, the program population was more diverse, with a much higher proportion of Hispanic (and Spanish-speaking) and African American patients than would have resulted only from the increased proportion of these groups in the state’s population during the same period: 29 and 2.5%, respectively [48, 49]. The program’s patients also tended to be less educated, less employed, in poorer physical and mental health, and were more likely to be living alone after the program redesign. It is not possible to link these changes specifically to the use of patient navigators, or even to the program’s expansion to include patients with chronic illnesses, but the changed demographics do indicate that the program’s social context differed markedly after the program change and as intended by those who designed it.

Given these challenges, the patients’ greater satisfaction with their overall health care after the program change is a very positive result, but this higher level of satisfaction was explained entirely by the associated differences in patient characteristics. In contrast, satisfaction declined with the clinic and the care provider, except for those who were similar in their health care needs to those in the program before its redesign—those who were eligible for case management services due to heightened cancer risk.

The higher levels of satisfaction with health care overall among the apparent first-generation immigrants was generally consistent with research that identifies particularly positive attitudes and behaviors in the first generation [50, 51], although the unique effect on clinic-specific satisfaction for Portuguese speakers may also indicate particularly positive features of the few health care sites that served most Portuguese speakers. The lower level of satisfaction among African American patients with their overall health care after the program redesign did not occur with the program-specific indicators, so it may indicate more severe chronic health problems or more general health care access problems rather than poorer program services per se.

The diminished program-related average satisfaction level after the redesign may reflect a change from the focus on successful resolution of cancer risk for most of the pre-change patients, as compared to the ongoing problems due to chronic illness of the patients who predominated after the program change. It is unlikely that many patients after the eligibility expansion found that their chronic health problems had been resolved by the program or its staff—or more generally by the health clinic from which they received their program services. This interpretation is strengthened by the finding that satisfaction with the patient navigator was higher than it was with the case manager for those patients who had both—in other words, patients enrolled after the program change who were being seen for cancer risk. This parallels the finding from the Patient Navigation Research Program of improved satisfaction due to patient navigation among socially disadvantaged patients with recent breast or colorectal cancer diagnoses [13, 14, 52].

Satisfaction with the program or clinic was uniquely influenced by health care barriers—specifically by more perceived economic as well as practical problems with obtaining care—as well as by less education, poorer self-rated health, and reports that high costs had deterred care in the past. In general, these results confirm Post et al.’s [11] finding of the selectivity of effects of perceived barriers on satisfaction. The lack of association of knowing they had a patient navigator with the satisfaction indicators suggests that there is room for strengthening the patient navigation program, perhaps by creating more opportunities for in-person contact [53, 54].

Older age and more perceived practical barriers were the only factors associated with less satisfaction across its multiple dimensions. The other factors associated with the different satisfaction indicators suggest that satisfaction with different aspects of health care are differentially susceptible to influence by patient background and current health problems. This indicates the importance for research on health care satisfaction of the use of satisfaction indicators with different levels of specificity, as emphasized first by Roberts, Pascoe, & Attkisson [20].

Our design had several limitations. First, a panel design was not possible because of the turnover in the original program’s patients as their cancer risks were identified. In addition, the rating of health care overall in the original program survey focused on health care services within the past 2 years, while the comparable question in the post-transition survey was narrowed to the past year because of the new program’s more limited history. The focus of the rating of satisfaction with the primary health care provider also had to shift from the case manager to the patient navigator, although our separate pre-post analysis of satisfaction just with the case manager for those who had a case manager after the program redesign—and our comparison of these patients’ satisfaction with their patient navigator to their satisfaction with their case manager—helps to clarify the effect of this shift in primary program delivery staff. In addition, only two questions about program satisfaction were repeated in the two surveys, resulting in a less robust index. It is also important to note that none of the apparent program effects on satisfaction were altered significantly by inclusion of the barriers indicators in the model, suggesting less tangible mediators of program effect that we did not capture [22].

The absence of a special overall satisfaction benefit of the program redesign for the more disadvantaged ethnic and linguistic groups [13, 14] may be a consequence of the limitation of our entire sample to patients in a program serving only socioeconomically disadvantaged patients. In fact, as already noted, the lower level of overall health care satisfaction after the program redesign among African American patients may reflect elevated chronic health problems in this disadvantaged group within the program after eligibility was expanded. By contrast, patient navigators did elicit higher satisfaction ratings than did case managers, when only those patients being seen for cancer risk were considered. Thus, even though we cannot be sure that the addition of patient navigators to the program workforce was responsible for the increased patient diversity, it is clear that the patient navigators were making a worthwhile contribution to serving the program’s patients.

The shift in focus of the original survey questions on satisfaction with the program itself to satisfaction with the clinic or health center where the program was implemented in the post-transition survey was necessitated by the less distinct program services delivered by patient navigators compared to those delivered by the case managers, but it means that the post-change patients could have been expressing satisfaction with a broader range of health care experiences at the health care location instead of just those delivered by program staff. Finally, although the difficulties our interviewers encountered in locating program patients are characteristic of attempts to survey lower SES groups, the obtained sample may as a result not represent the experience of all program patients. Thus, while our findings pertain to a range of issues that recur in the health care system, they must be applied to different contexts with caution.

## Conclusions

The addition of patient navigators to the case management program was a key element in organizational changes designed to lower access barriers so as to reach more of the underserved population. At the same time, the more holistic focus on managing chronic illness created greater challenges for delivery of satisfying health services. While in the original program, the large majority of patients received a definitive diagnosis of the absence of cancer and so had this health concern relieved, those with chronic illnesses seen after the program redesign would have experienced no such definitive resolution of a health concern; instead, they would face ongoing needs for care, perhaps accompanied by a need for behavioral change.

Recognizing the importance of patient satisfaction must be balanced by understanding its complexity. Our analysis of the apparent effects on satisfaction of the redesign of the Massachusetts NBCCEDP program makes it clear that a single-minded focus on maximizing patient satisfaction can both obscure the effects of an intervention and deter more ambitious efforts to improve delivery of health care. As expected, the patient navigators seemed to increase the satisfaction of patients like those served in the original program with the program itself, while the patient navigators for these same patients received higher satisfaction scores than did case managers in the previous program. However, these effects were obscured until the patients being seen for cancer risk were distinguished from those being seen for chronic illness. In addition, an apparent positive effect of the redesigned program on overall health care satisfaction actually reflected a change in the orientations of new patients from different backgrounds.

We do not conclude that patient navigators should only be used to enhance cancer care, but rather that their role and expectations for their performance need to be tailored to the type of health care program in which they work. The cancer care program we studied was extended because physicians on our Expert Panel had identified chronic illness as lowering rates of diagnostic testing; the finding that this broader focus was associated with lower satisfaction ratings indicates only how much more challenging it is. Our results also suggest that more attention is needed to the mechanism by which patient navigators may improve patient satisfaction. While the apparent patient navigator effect we identified was not explained by a reduction of any perceived barriers, we suggest that the interpersonal connection between patient navigators and patients may be what is most important.

The program redesign to serve a needier patient population can be judged a success on its own terms, even though it resulted in lower average levels of satisfaction with the program and its primary staff. Our research thus highlights the potential of and challenges for health care delivery systems seeking to broaden program participation and set more ambitious goals for health improvement.

## Supplementary information

**Additional file 1.** Women’s Health Network Survey. Questionnaire for survey of Women’s Health Network patients (pre-survey).

**Additional file 2.** Care Coordination Program Survey. Questionnaire for survey of Care Coordination Program patients (post-survey).

## Data Availability

The datasets generated and analysed during the study are not publicly available due to the terms of the state agency contract under which the data were collected as part of a state program evaluation, but are available from the corresponding author on reasonable request.

## Abbreviations

CCP: Care Coordination Program (Massachusetts)
CDC: Centers for Disease Control and Prevention
CM: Case Manager
DPH: Department of Public Health (Massachusetts)
IRB: Institutional Review Board (University of Massachusetts Boston)
NBCCEDP: National Breast and Cervical Cancer Early Detection Program
OLS: Ordinary Least Squares (regression analysis)
PN: Patient Navigator
R^2^: Coefficient of Determination
WHN: Women’s Health Network (Massachusetts)
