# Supplementary material for: Increasing health service access by expanding disease coverage and adding patient navigation: challenges for patient satisfaction
Source: BMC Health Serv Res. 2020 Mar 6;20:175. doi: 10.1186/s12913-020-5009-x (PMC7059366; doi:10.1186/s12913-020-5009-x)
Supplement: Supplementary file 1 — Additional file 1. Women’s Health Network Survey. Questionnaire for survey of Women’s Health Network patients (pre-survey). [file 12913_2020_5009_MOESM1_ESM.pdf]

To begin, I would like to ask you a few questions about your general health and how you get health care.

1. Would you say that in general your health is excellent, very good, good, fair or poor?

Excellent	[ ]
Very Good	[ ]
Good	[ ]
Fair	[ ]
Poor	[ ]

2. Have you had any physical health problems in the past year?

Yes	[ ]
No	[ ] {Skip to Q2b}
Don't Know	[ ] {Skip to Q2b}

2a. Did these health problems affect, in any way, your ability to carry out your normal daily activities?

Yes	[ ]
No	[ ]

2b. During the past month, have you been bothered by feeling down, depressed or hopeless?

Yes	[ ]
No	[ ]

2c. During the past month, have you been bothered by having little interest or pleasure in doing things?

Yes	[ ]
No	[ ]

3. Do you currently have any kind of health insurance coverage at all?

Yes	[ ] {Skip to Q6}
No	[ ]

4. Do you currently have any health insurance coverage through government programs such as Medicare, Medicaid, or MassHealth?

Yes	[ ] {Skip to Q6}
No	[ ]

5. So, you currently do NOT have ANY health insurance coverage at all. Is that correct?

Yes	[ ]
No	[ ]

6. In the past 2 years, has there been anytime when you needed to get medical care but could not because of the cost?

Yes	[ ]
No	[ ]
Don't Know	[ ]

7. Do you have a place, such as a doctor's office or clinic, where you usually go for medical care?

Yes	[ ]
No	[ ]
Don't Know	[ ]

8. Do you have one person who you think of as your personal doctor or health care provider?

Yes	[ ] {Skip to Q9}
No	[ ]
Don't Know	[ ]

8a. Do you have more than one person who you think of as your doctor or health care provider?

Yes	[ ]
No	[ ]

9. Now I would like you to think about all the health care you have received in the past two years. On a scale from 0 to 10, where 0 is the worst health care possible and 10 is the best health care possible, how would you rate the overall quality of the health care that you have received in the past two years?

Numerical answer: _______________

These next questions ask about your experiences with breast and cervical cancer screening and testing.

10. A mammogram is an X-Ray of each breast to look for breast cancer. Have you ever had a mammogram?

Yes [ ]
No [ ] {Skip to Q12}
Don't Know [ ] {Skip to Q12}

11. In what year did you have your most recent mammogram?

Year: ______________

Don't Know [ ]

12. A clinical breast exam is when a doctor, nurse, or other health professional feels the breasts for lumps. Have you ever had a clinical breast exam?

Yes [ ]
No [ ] {Skip to Q14}
Don't Know [ ] {Skip to Q14}

13. In what year did you have your most recent clinical breast exam?

Year: ______________

Don't Know [ ]

14. A Pap test is a test for cancer of the cervix. Have you ever had a Pap test?

Yes [ ]
No [ ] {Skip to Q16}
Don't Know [ ] {Skip to Q16}

15. In what year did you have your most recent Pap test?

Year: _____________

Don't Know [ ]

16. The Women's Health Network is a program sponsored by the Department of Public Health. The program pays for free breast and cervical cancer screening and helps women who need follow up care. The Women's Health Network services are provided through community health centers, hospitals, visiting nurse services and doctor's offices.

Have you ever heard of the Women's Health Network?

Yes	[ ]
No	[ ] {Skip to Q18}
Don't Know	[ ] {Skip to Q18}

[IF Q10, Q12 AND Q14 ARE ALL NO, THEN SKIP TO Q65]
17. As far as you know, have you ever received help getting screening or testing for breast or cervical cancer from the Women's Health Network?

Yes	[ ] {Skip to Q19}
No	[ ]
Don't Know	[ ]

18. Did anyone from the [site name] ever help you get screening or testing for breast or cervical cancer?

Yes	[ ]
No	[ ]
Don't Know	[ ]

19. Have you ever been told, or informed in any way, that you need further testing after a mammogram, clinical breast exam or Pap test?

Yes	[ ]
No	[ ] {Skip to Q65}
Don't Know	[ ]

[Under the Women's Health Network,] when a woman is screened for breast or cervical cancer and if the results show that she needs follow-up testing or treatment, a health professional is assigned to help her get those services. The health professional may be called a case manager and often works with a program coordinator or client navigator. I would like to ask you about services you may have received from any of these health professionals. For the remainder of this interview, I will simply refer to this health professional who helped you as a case manager.

[The bracketed phrase above is included only if respondent has indicated getting help from the Women's Health Network, that is Q17 is YES]

20. Has a case manager helped you in any way to get follow-up testing or treatment for breast or cervical cancer?

Yes	[ ]
No	[ ] {Skip to Q30}

20a. Has a case manager helped you get follow-up testing for breast cancer, cervical cancer or both?

Breast cancer	[ ]
Cervical cancer	[ ]
Both	[ ]

21. When did this case manager <u>most recently</u> help you get this follow-up testing, in the past six months or more than 6 months ago?

In the past 6 months	[ ]
6 or more months ago	[ ] {Skip to Q21b}

21a. When did this case manager <u>most recently</u> help you get this follow-up testing, in the past week, at least one week but less than one month ago, at least one month but less than three months ago, or three months ago or longer?

In the last week	[ ] {Skip to Q22}
At least 1 week but less than 1 month ago	[ ] {Skip to Q22}
At least 1 month but less than 3 months ago	[ ] {Skip to Q22}
3 months ago or longer	[ ] {Skip to Q22}
Don't Know	[ ] {Skip to Q22}

21b. When did this case manager <u>most recently</u> help you get this follow-up testing, at least six months but less than one year ago, at least one year but less than two years ago or two or more years ago?

At least 6 months but less than 1 year ago	[ ]
At least 1 year but less than 2 years ago	[ ]
2 or more years ago	[ ]
Don't Know	[ ]

22. How much help have you received in getting follow-up testing for breast or cervical cancer? Would you say that you have received no help, a little help or a lot of help?

No help	[ ]
A little help	[ ]
A lot of help	[ ]

23. Did the case manager describe to you the types of follow-up tests that would be required?

Yes	[ ]
No	[ ]
Don’t Know	[ ]

24. Did the case manager describe the results that might come about from these follow-up tests?

Yes	[ ]
No	[ ]
Don’ Know	[ ]

25. How often did the case manager explain follow-up testing services in a way you could understand? Would you say always, usually, sometimes, rarely or never?

Always	[ ]
Usually	[ ]
Sometimes	[ ]
Rarely	[ ]
Never	[ ]

26. How often did the case manager listen carefully to your questions or concerns? Would you say always, usually, sometimes rarely or never?

Always	[ ]
Usually	[ ]
Sometimes	[ ]
Rarely	[ ]
Never	[ ]

27. How often did the case manager treat you with respect? Would you say always, usually sometimes, rarely or never?

Always	[ ]
Usually	[ ]
Sometimes	[ ]
Rarely	[ ]
Never	[ ]

28. How often did the case manager spend enough time with you? Would you say always, usually, sometimes, rarely or never?

Always	[ ]
Usually	[ ]
Sometimes	[ ]
Rarely	[ ]
Never	[ ]

29. Using a scale from 0-10, where 0 is the worst possible help and 10 is the best possible help, how would you rate the help you received from your case manager?

Numerical Answer: __________

[IF Q20A IS BREAST CANCER OR BOTH, ASK Q30, ELSE SKIP TO Q35]

30. At this time, are you waiting to have additional tests for breast cancer?

Yes	[ ]
No	[ ]
Don't Know	[ ]

31. Did you <u>ever</u> receive results that tell you that you <u>have</u> breast cancer?

Yes	[ ]
No	[ ] {Skip to INTCHK1}
Don't Know	[ ]

32. At this time, are you waiting to begin treatment for breast cancer?

Yes	[ ] {Skip to INTCHK1}
No	[ ]
Don't Know	[ ]

33. At this time, have you already begun treatment for breast cancer?

Yes [ ]
No [ ] {Skip to INTCHK1}
Don't Know [ ]

34. At this time, have you completed treatment for breast cancer?

Yes [ ]
No [ ]
Don't Know [ ]

34a. At this time, have your most recent tests indicated that you do have breast cancer?

Yes [ ]
No [ ]
Don't Know [ ]

[INTCHK1: IF Q20A IS BREAST CANCER ONLY, SKIP TO INTCHK2]

35. At this time, are you waiting to have additional tests for cervical cancer?

Yes [ ]
No [ ]
Don't Know [ ]

36. Did you <u>ever</u> receive results that tell you that you <u>have</u> cervical cancer?

Yes [ ]
No [ ] {Skip to INTCHK2}
Don't Know [ ]

37. At this time, are you waiting to begin treatment for cervical cancer?

Yes [ ] {Skip to INTCHK2}
No [ ]
Don't Know [ ]

38. At this time, have you already begun treatment for cervical cancer?

Yes [ ]
No [ ] {Skip to INTCHK2}
Don't Know [ ]

39. At this time, have you completed treatment for cervical cancer?

Yes [ ]
No [ ]
Don't Know [ ]

39a. At this time, have your most recent results indicated that you do have cervical cancer?

Yes [ ]
No [ ]
Don't Know [ ]

***[INTCHK2: If Q17 is YES, then read]* Now I will ask you questions about services that you may have received from the Women's Health Network case manager.**
***[If Q17 is NO or DON'T KNOW and Q20 is YES, then read]* Now I will ask you questions about services that you may have received from a case manager.**
[If Q17 is NO or DON'T KNOW and Q20 is NO, then read} Now I will ask you a few questions about services that you may have received.
[If Q17 is NO or DON'T KNOW and Q20 is NO, then replace 'the case manager' with 'any health professional' in Q40, Q41, Q42, Q43 and Q44]

40. Did the case manager refer you to a place to go to get your follow-up tests?

Yes [ ]
No [ ] {Skip to Q41}

40a. How satisfied were you with the referral you received? Would you say very satisfied, satisfied, dissatisfied or very dissatisfied?

Very satisfied [ ]
Satisfied [ ]
Dissatisfied [ ]
Very dissatisfied [ ]

41. Did the case manager provide you with information about the details of any [breast or cervical cancer] follow-up testing that you needed?

Yes [ ]
No [ ] {Skip to Q42}

41a. How satisfied were you with the information on follow-up testing you received? Would you say very satisfied, satisfied, dissatisfied or very dissatisfied?

Very Satisfied [ ]
Satisfied [ ]
Dissatisfied [ ]
Very Dissatisfied [ ]

42. Did the case manager provide you with information about the cost of the [breast or cervical cancer] follow-up testing you needed?

Yes [ ]
No [ ] {Skip to Q43}

42aa. Did you have to pay any of the costs for the [breast or cervical cancer] follow-up testing?

Yes [ ]
No [ ] {Skip to Q42b}
Don't Know [ ]

42a. How difficult was it for you to pay for any follow-up testing? Would you say very difficult, somewhat difficult, a little difficult or not difficult at all?

Very difficult [ ]
Somewhat difficult [ ]
A little difficult [ ]
Not difficult at all [ ]

42b. How satisfied were you with the accuracy of the cost information you received? Would you say very satisfied, satisfied, dissatisfied or very dissatisfied?

Very Satisfied [ ]
Satisfied [ ]
Dissatisfied [ ]
Very Dissatisfied [ ]

43. Did the case manager provide you with information about the details of the [breast or cervical cancer] treatment that you may have been scheduled to have?

Yes [ ]
No [ ] {Skip to Q44}

43a. How satisfied were you with the information you received? Would you say very satisfied, satisfied, dissatisfied or very dissatisfied?

Very Satisfied [ ]
Satisfied [ ]
Dissatisfied [ ]
Very Dissatisfied [ ]

44. Did the case manager provide you with information about the cost of the [breast or cervical cancer] treatment you may have been scheduled to have?

Yes [ ]
No [ ] {Skip to INTCHK3}

44aa. Did you have to pay any of the costs for the [breast or cervical cancer] treatment?

Yes [ ]
No [ ] {Skip to Q44b}
Don't Know [ ]

44a. How difficult was it for you to pay for the [breast or cervical cancer] treatment? Would you say very difficult, somewhat difficult, a little difficult, or not difficult at all?

Very difficult [ ]
Somewhat difficult [ ]
A little difficult [ ]
Not difficult at all [ ]

44b. How satisfied were you with the accuracy of the cost information you received? Would you say very satisfied, satisfied, dissatisfied or very dissatisfied?

Very Satisfied [ ]
Satisfied [ ]
Dissatisfied [ ]
Very Dissatisfied [ ]

***[INTCHK3: If Q17 is YES, then read] The next questions ask about your relationship with the* Women's Health Network case manager who was providing you with these services.**
***[If Q17 is NO or DON'T KNOW and Q20 is YES, then read] The next questions ask about your relationship with the* case manager who was providing you with these services.**
[If Q17 is NO or DON'T KNOW and Q20 is NO, then read} The next questions ask about your relationship with the person who was providing you with these services

45. How satisfied were you with the manner in which the case manager or health professional (READ LIST)? Would you say that you were very satisfied, satisfied, dissatisfied or very dissatisfied?

	Very Satisfied	Satisfied	Dissatisfied	Very Dissatisfied
a. kept in touch with you during the time of your testing and treatment?	[ ]	[ ]	[ ]	[ ]
b. obtained your consent for all your procedures?	[ ]	[ ]	[ ]	[ ]
c. respected the privacy of your medical records?	[ ]	[ ]	[ ]	[ ]
d. helped you to communicate with other health care providers?	[ ]	[ ]	[ ]	[ ]
e. provided you with a private place and time to talk to you?	[ ]	[ ]	[ ]	[ ]
f. was sensitive to your needs and background?	[ ]	[ ]	[ ]	[ ]
g. was knowledgeable in answering any questions you had?	[ ]	[ ]	[ ]	[ ]

Now I am going to read a list of potential barriers to receiving breast or cervical cancer screening, testing, or treatment. For each, I'd like you to tell me how much of a problem it has been for you.

How much of a problem has (READ LIST) been for you in getting the screening, testing or treatment for breast or cervical cancer that you required? Would you say a major problem, moderate problem, minor problem or no problem at all?

{Questions 47 and 48 are not asked if the person lists English as their primary language}
{Question 56 has an answer option of "did not have health insurance"}

	Major Problem	Moderate Problem	Minor Problem	No Problem At All
46. transportation to or from clinics or other facilities	1	2	3	4
47. communicating with health care staff in English	1	2	3	4
48. reading English	1	2	3	4
49. your knowledge about the healthcare system	1	2	3	4
50. scheduling appointments	1	2	3	4
51. concern about medical bills you may have to pay	1	2	3	4
52. other health problems you have	1	2	3	4
53. the attitudes of family or friends	1	2	3	4
54. fear of receiving bad news	1	2	3	4
55. fear of pain due to testing or treatment	1	2	3	4
56. dealing with your health insurance	1	2	3	4
57. your citizen or immigration status	1	2	3	4
58. your distrust of the health care system	1	2	3	4
59. your desire for some other type of treatment	1	2	3	4
60. having the time needed to have the tests and treatments	1	2	3	4

[If Q16 is NO or DON'T KNOW, Skip to Q65]

These next questions are about your overall impressions of the Women's Health Network program. I am going to read a list of statements. For each statement, please tell me whether you Strongly Agree, Agree, Disagree or Strongly Disagree.

	Strongly Agree	Agree	Disagree	Strongly Disagree
61. I would recommend this program to a friend who has similar needs.	1	2	3	4
62. The location of this program is convenient.	1	2	3	4
63. Overall, I am satisfied with this program.	1	2	3	4
64. In general, I am doing better because of this program.	1	2	3	4

We are almost done. The last few questions are about your personal situation.

65. Are you currently married, separated, divorced, widowed or have you never been married?

Married	[ ]
Separated	[ ]
Divorced	[ ]
Widowed	[ ]
Never Married	[ ]

{If Q65 is anything but "Married", do not ask Q66b}
66. Please answer yes or no to each of the following questions. Do you currently live with…

	YES	NO	
a. no one else, that is, you live alone?	[ ]	[ ]	{If YES, Skip to Q68}
b. a husband or wife?	[ ]	[ ]	{If YES, Skip to Q66d}
c. a partner?	[ ]	[ ]	
d. other adult relatives?	[ ]	[ ]	
e. your children?	[ ]	[ ]	
f. children that are not yours?	[ ]	[ ]	
g. friends?	[ ]	[ ]	
h. roommates?	[ ]	[ ]	
i. someone else?	[ ]	[ ]	

67. Are you currently working at a full-time job, a part time job, or not working at all?

Full-time job [ ] {Skip to Q70}
Part-time job [ ] {Skip to Q70}
Not working [ ]

68. Are you currently looking for work?

Yes [ ]
No [ ]

69. Are you currently retired?

Yes [ ]
No [ ]

70. Are you currently a full-time student, a part-time student or not a student at all?

Full Time Student [ ]
Part Time Student [ ]
Not a Student [ ]

Thank you for your time and cooperation.
