# Supplementary material for: Increasing health service access by expanding disease coverage and adding patient navigation: challenges for patient satisfaction
Source: BMC Health Serv Res. 2020 Mar 6;20:175. doi: 10.1186/s12913-020-5009-x (PMC7059366; doi:10.1186/s12913-020-5009-x)
Supplement: Supplementary file 2 — Additional file 2. Care Coordination Program Survey. Questionnaire for survey of Care Coordination Program patients (post-survey). [file 12913_2020_5009_MOESM2_ESM.pdf]

PATIENT NAVIGATOR CLIENT QUESTIONNAIRE

To begin, I would like to ask you a few questions about how you get medical care.

S0a. Do you have a place, such as a doctor's office or clinic, where you usually go for medical care?

1 YES

5 NO

7 Don't Know

S0b. Do you have one person who you think of as your doctor or primary health care provider?

1 YES

5 NO {SKIP TO S0c.}

7 Don't Know {SKIP TO S0c.}

S0b1. Is that person a …{READ EACH}?

1 DOCTOR	YES	NO	NA
2 NURSE PRACTITIONER	YES	NO	NA
3 NURSE	YES	NO	NA
4 CASE MANAGER	YES	NO	NA
5 SOME OTHER PROVIDER	YES__{SPECIFY}	NO	NA

(ALL SKIP TO S1)

S0c. Do you have more than one person who you think of as your doctor or health care provider?

1 YES
2 NO {SKIP TO S1.}

S0c1. Are any of these other people …{READ EACH}?

1 DOCTOR	YES	NO	NA
2 NURSE PRACTITIONER	YES	NO	NA
3 NURSE	YES	NO	NA
4 CASE MANAGER	YES	NO	NA
5 SOME OTHER PROVIDER	YES__{SPECIFY}	NO	NA

S1. In the last year, have you received any medical care from {HEALTH CLINIC}?

1 YES

5 NO [IF NO, TERMINATE INTERVIEW]

S1a. Is this the place where you usually go for medical care?

1 YES

5 NO

9 NA

S1b.In the past year, have you gone to any other place to get medical care, such as to another clinic or to a private doctor?

1 YES

5 NO {skip to S2}

9 NA

S1c. Thinking about all the medical care you have received in the last year, about how much of that care have you received from {HEALTH CLINIC}?

1 ALL MEDICAL CARE FROM {HEALTH CLINIC}

2 MOST

3 SOME

4 A LITTLE

5 NONE **[IF NONE, TERMINATE INTERVIEW]**

9 NA

(IF SITE HAS 0 OR MORE THAN 4 PN'S SKIP TO S2a)
S2. In the last year, have you received any help from {PT NAVIGATOR NAME OR PN2 OR PN3 or PN4}?

1 YES{ASK FOR NAMES AND RECORD}{SKIP TO S2CHECK}

5 NO

9 NA

S2a. In the last year, have you received any help from a health care professional or professionals at {HEALTH CLINIC}, who are sometimes called case managers, or patient navigators, or patient coordinators?

1 YES {ASK FOR NAMES AND RECORD}

5 NO **{SKIP TO A1}**

9 NA

S2check: HOW MANY NAMES IDENTIFIED

1 ONE PN {SKIP TO S3}
2 MORE THAN ONE PN IDENTIFIED

S2a. Who is the PN that you had the **most contact** with in the past year?

1 ONE NAME SPECIFIED {______________________________________}{skip to S3}

2 ALL EQUAL

S2a1. Who is the PN that you had most **recent** contact with?

1 NAME SPECIFIED {______________________________________}

S3. Now I'd like to ask a few questions about {PN WITH MOST CONTACT; OR PN WITH MOST RECENT CONTACT}

In the last year, has {PT NAVIGATOR NAME} ever talked to you by phone or in person?

1 PHONE

2 IN PERSON {SKIP to S3b.}

3 BOTH BY PHONE AND IN PERSON

5 NO {SKIP TO A1}

9 NA {SKIP TO A1}

S3a. In the last year, how often did {PN} TALK TO YOU BY PHONE?

1 1 TIME {IF S3=3 GOTO S3b ELSE SKIP TO S4.}

2 2-4 TIMES { IF S3=3 GOTO S3b ELSE SKIP TO S4.}

3 5-10 TIMES { IF S3=3 GOTO S3b ELSE SKIP TO S4.}

4 11 OR MORE TIMES { IF S3=3 GOTO S3b ELSE SKIP TO S4.}

5 DK { IF S3=3 GOTO S3b ELSE SKIP TO S4.}

S3b. In the last year, how often did {PN} TALK TO YOU IN PERSON?

1 1 TIME

2 2-4 TIMES

3 5-10 TIMES

4 11 OR MORE TIMES

5 DK

S4. In what way has {PT NAVIGATOR NAME} helped you? {READ EACH and CHECK ALL THAT APPLY}

A.	Served as my interpreter	YES	NO	NA
B.	Helped find an interpreter	YES	NO	NA
C.	Helped with transportation	YES	NO	NA
D.	Helped find resources in the community	YES	NO	NA
E.	Helped with health insurance	YES	NO	NA
F.	Helped with scheduling appointments	YES	NO	NA
G.	Attended appointments with me	YES	NO	NA
H.	Helped find information about medical issues, including tests	YES	NO	NA
I.	Was someone to talk with about your medical problems or condition	YES	NO	NA
J.	Helped me get connected to my doctor in an emergency	YES	NO	NA
K.	Other	YES ______________________________	NO	NA

Now, I would like to ask you a few questions about your background.

A1. In what country were you born?

1 UNITED STATES

2 SOUTH AMERICA {specify country____________________}

3 CENTRAL AMERICA {specify country ____________________}

4 CARIBBEAN ISLANDS {specify country ____________________}

5 OTHER {specify country ____________________}

9 NA

A1a. Are you of Spanish, Hispanic or Portuguese descent?

1 YES

5 NO {skip to A1c}

7 DON'T KNOW

9 NA {skip to A1c}

A1b. What ethnic group or nationality do you consider yourself? (IF NEEDED: MEXICAN, CHICANO, PUERTO RICAN, CUBAN, BRAZILIAN, CAPE VERDEAN?)

(MARK ALL MENTIONED) {PROGRAM TO TAKE UP TO 3 MENTIONED}

1 MEXICAN

2 CHICANO

3 PUERTO RICAN

4 CUBAN

5 OTHER SPANISH (SPECIFY) ______________________________

6 BRAZILIAN

7 CAPE VERDEAN

8 OTHER PORTUGUESE (SPECIFY) __________________________

97 DON'T KNOW

99 NA

A1c. (IF A1a = YES) In addition to being Spanish/Hispanic/Portuguese, (ENDIF) What race do you consider yourself? (READ EACH)

1 White or Caucasian YES NO NA

2 Black or African American YES NO NA

3 Asian (Asian Indian, Chinese, Filipino, Japanese, Korean, Vietnamese) YES NO NA

4 Native Hawaiian or Pacific Islander (Guamanian or Chamorro, Samoan) YES NO NA

5 American Indian or Alaska Native YES NO NA

7 Some other race YES (specify): NO NA

9 NA

A2. (IF A1 =1 US skip to A3) In what year did you first come to live in the United States?

— — — —

A3a. How many of your parents were born in the U.S.?

0 NONE

1 ONE

2 TWO

7 DON'T KNOW

9 NA

A3b. How many of your grandparents were born in the U.S.?

0 NONE

1 - 4 ENTER NUMBER _____

7 DON'T KNOW

9 NA

A4. Is English your native language?

1 YES

2 NO

3 "BILINGUAL"

7 DK

9 NA

A5. What language do you mainly speak?

1. ENGLISH
2. SPANISH
3. PORTUGUESE
4. HAITIAN/CREOLE
5. OTHER LANGUAGE _______________
9. NA

A6. *(IF A4=YES & A5=English skip to A10)*

What language do you speak at home?

1. ENGLISH
2. SPANISH
3. PORTUGUESE
4. HAITIAN/CREOLE
5. OTHER LANGUAGE _______________
9. NA

A7. *(IF A5 & A6=English skip to A10)*

How well would you say you speak English? Would you say …

1 VERY WELL

2 WELL

3 NOT WELL

4 NOT AT ALL

9 NA

A8. How well would you say you read English? Would you say …

1 VERY WELL

2 WELL

3 NOT WELL

5 NOT AT ALL

9 NA

A9. How well would you say you write English?

1 VERY WELL

2 WELL

3 NOT WELL

4 NOT AT ALL

9 NA

A10. How important are your ethnic or national origin, tradition and values in the decisions you make about your health care? Would you say very important, somewhat important, not very important, or not at all important?

1 VERY IMPORTANT

2 SOMEWHAT IMPORTANT

3 NOT VERY IMPORTANT

4 NOT AT ALL IMPORTANT

9 NA

A11. How much would you say a health care provider like a doctor or nurse needs to understand your culture to be able to provide you with the best health care possible? Would you say a lot, somewhat, a little, or not at all?

1 A LOT

2 SOMEWHAT

3 A LITTLE

4 NOT AT ALL

9 NA

(IF A4=YES OR A5 = ENGLISH SKIP TO B1)

A12a. Do you have any health care providers who do not speak your native language?

1 YES

2 NO (skip to B1)

9 NA (skip to B1)

A12b. When you talk to a health care provider like a doctor or nurse who does not speak your native language, do you usually need an interpreter?

1 YES

2 NO (skip to B1)

9 NA (skip to B1)

A13. In the last year, have you used any of the following people as interpreters when speaking to any health care provider?

1	HEALTH CLINIC'S INTERPRETER	YES	NO
2	PATIENT NAVIGATOR [NAME]	YES	NO
3	PHONE INTERPRETATION SERVICE	YES	NO
4	FAMILY MEMBER	YES	NO
5	SOMEONE ELSE {PLEASE DESCRIBE}	YES	NO
9	NA (skip to A18b)		

(IF A13=1, ASK A13A; OTHERWISE SKIP)

A13a. How often do you use {HEALTH CLINIC'S} interpreters- all the time, some of the time, or very little of the time?

1	ALL THE TIME	(skip to B1)
2	SOME OF THE TIME	(skip to B1)
3	VERY LITTLE OF THE TIME	(skip to B1)
9	NA	(skip to B1)

A13b. Would you like to have an interpreter available to you when you speak with a health care provider like a doctor or nurse or case manager?

1 YES

2 NO

IntroB1. Now, I would like to ask you some questions about your general health.

B1. Would you say that in general your health is excellent, very good, good, fair or poor?

1 Excellent
2 Very Good
3 Good
4 Fair
5 Poor

(SKIPPING THIS QUESTION FOR ALL, Feb. 1)
B2. Have you had any physical health problems in the past year?

1 YES

2 NO {Skip to B2b}

7 DON'T KNOW{Skip to B2b}

B2a. Have you had any of the following in the past year?

1	ASTHMA	YES	NO	NA
2	CANCER CONCERNS	YES	NO	NA
3	CARDIOVASCULAR DISEASE (CVD)	YES	NO	NA
4	HYPERTENSION OR HIGH BLOOD PRESSURE	YES	NO	NA
5	DIABETES	YES	NO	NA
6	DEPRESSION OR ANXIETY	YES	NO	NA
7	SUBSTANCE ABUSE ISSUES	YES	NO	NA
8	DENTAL PROBLEMS	YES	NO	NA
9	VISION PROBLEMS	YES	NO	NA
10	OTHER ISSUES (What are they?_____)			
11	Have you been Smoking on a regular basis?	YES	NO	NA

B2a1. Did these health problems affect, in any way, your ability to carry out your normal daily activities?

1 YES

2 NO

B2aa. Did you use any of the following health care services in the past year?

{READ and CHECK ALL THAT APPLY}

1	HOSPITALIZATION (OVER NIGHT)	YES	NO	NA
2	SMOKING CESSATION PROGRAM	YES	NO	NA
3	BEHAVIORAL HEALTH PROGRAM, THERAPY	YES	NO	NA
4	NUTRITIONIST, NUTRITION OR OR COOKING CLASSES	YES	NO	NA
5	DENTAL CARE	YES	NO	NA
6	EMERGENCY SERVICES			
7	OTHER HEALTH CARE SERVICES (specify) ______________________			

B2ab.Overall, do you feel that there has been any change in your health problems in the last year? Would you say that the problems ….

1 have gotten better
2 have stayed about the same
3 have gotten worse?

4 (IF NEEDED) SOME PROBLEMS HAVE GOTTEN BETTER, SOME WORSE
9 NA

B2b. During the last two weeks, have you been bothered by feeling down, depressed or hopeless?

1 NOT AT ALL
2 SEVERAL DAYS
3 MORE THAN HALF THE DAYS
4 NEARLY EVERY DAY

B2c. During the last two weeks, have you been bothered by having little interest or pleasure in doing things?

1 NOT AT ALL
2 SEVERAL DAYS
3 MORE THAN HALF THE DAYS
4 NEARLY EVERY DAY

B3. I would like to ask you a few questions about health care coverage you may use to pay for most of your medical care.

Do you currently have health care coverage that you get… [IF YES TO ANY QUESTION, SKIP OUT OF THE REST OF THE SERIES]

a. through your or someone else's employer?

b. through MassHealth or Medicaid, this includes CommonHealth or MassHealth HMO's offered through Neighborhood Health Plan, Fallon Community Health Plan, BMC HealthNet or Network Health?

c. through any other type of public insurance such as Commonwealth Care, which provides either no-cost or subsidized care and is obtained through the Commonwealth Connector?

d. through Medicare?

e. by you or someone else buying it totally on your own?

f. some other way such as through the military, the VA or the Indian Health Service?

B4. [IF THE ANSWER IS NO TO ALL OF A. THROUGH F., THEN ASK:] So you currently do NOT have ANY health insurance coverage at all. Is this correct?

1 Yes
2 No B4a. If No, ask: What health insurance do you have? ________________
7 DON'T KNOW

B5. In the past year, has there been any time when you needed to get medical care but could not because of the cost?

1 YES
2 NO
7 DON'T KNOW

B6. Now I would like you to think about all the health care you have received from {HEALTH CLINIC} in the past year. On a scale from 0 to 10, where 0 is the worst health care possible and 10 is the best health care possible, how would you rate the overall quality of the health care that you have received in the past year?

Numerical answer: _______________

C intro. The next questions ask about your opinion. There are no right or wrong answers.

C1. How confident are you in your ability to make good decisions about your medical care…

1 Very confident
2 Somewhat confident
3 Not very confident
4 Not at all confident
9 NA

C2. How confident are you in your ability to make good decisions in general…

1 Very confident
2 Somewhat confident
3 Not very confident
4 Not at all confident
9 NA

(IF A4=YES OR A5 = ENGLISH SKIP TO…)

C3. When decisions have been made about health services you would get, did you have any difficulty speaking with or understanding the health care provider because you speak a different language?

1 YES
2 NO
9 NA

(ASK IF C3 = YES)

C3a. How big a problem was it for you? Would you say it was a big problem, moderate problem, small problem, or no problem at all?

1 BIG PROBLEM
2 MODERATE PROBLEM
3 SMALL PROBLEM
4 NO PROBLEM AT ALL
9 NA

[IF S2=NO SKIP TO C5]
C4. How satisfied were you with the manner in which the **patient navigator** (NAME) (READ LIST)? Would you say that you were very satisfied, satisfied, dissatisfied or very dissatisfied?

	Very Satisfied	Satisfied	Dissatisfied	Very Dissatisfied	Not Applicable /PN didn't do
a. kept in touch with you about your health care needs?	[ ]	[ ]	[ ]	[ ]	
b. helped you find resources in the community	[ ]	[ ]	[ ]	[ ]	
c. helped you to communicate with other health care providers?	[ ]	[ ]	[ ]	[ ]	
d. helped you with transportation	[ ]	[ ]	[ ]	[ ]	
e. helped you with scheduling appointments	[ ]	[ ]	[ ]	[ ]	

[IF CN FLAG OR S0b1 or S0c1]
C5. When a (IF GENDER IS FEMALE) woman (ELSE) man (ENDIF) is screened at [THE MEDICAL CENTER] for (IF GENDER IS FEMALE) breast, cervical or colon cancer (ELSE) prostate or colon cancer (ENDIF) and the results show that she/he needs follow-up testing or treatment, a health professional called a "case manager" may be assigned to help her/him get that testing or treatment. XXX [from S2] is a case manager at [THE MEDICAL CENTER], and the next questions are about her.

How satisfied were you with the manner in which the **case manager** (NAME) (READ LIST)? Would you say that you were very satisfied, satisfied, dissatisfied or very dissatisfied?

	Very Satisfied	Satisfied	Dissatisfied	Very Dissatisfied	Not Applicable /PN didn't do
a. kept in touch with you about your health care needs?	[ ]	[ ]	[ ]	[ ]	
b. helped me find resources in the community	[ ]	[ ]	[ ]	[ ]	
c. helped you to communicate with other health care providers?	[ ]	[ ]	[ ]	[ ]	
d. helped you with transportation	[ ]	[ ]	[ ]	[ ]	
e. helped you with scheduling appointments	[ ]	[ ]	[ ]	[ ]	
k. helped you find information about medical issues, including tests	[ ]	[ ]	[ ]	[ ]	

C6. Now I am going to read a list of potential barriers to receiving **health care**. For each, I'd like you to tell me how much of a problem it has been for you.

How much of a problem has (READ LIST) been for you in getting **health care** that you required? Would you say a major problem, moderate problem, minor problem or no problem at all?

{Questions b. and c. are not asked if the person lists English as their primary language}
{Question k. has an answer option of "did not have health insurance"}
{Skip Question l. if A1=1 (born in U.S.}

	Major Problem	Sometimes a Problem	Minor Problem	No Problem At All	NA
a. transportation to or from clinics or other facilities	1	2	3	4	9
b. communicating with health care staff in English	1	2	3	4	9
c. reading English	1	2	3	4	9
d. your knowledge about the healthcare system	1	2	3	4	9
e. scheduling appointments	1	2	3	4	9
f. concern about medical bills you may have to pay	1	2	3	4	9
g. other health problems you have	1	2	3	4	9
h. the attitudes of family or friends	1	2	3	4	9
i. fear of receiving bad news	1	2	3	4	9
j. fear or anxiety about testing or treatment	1	2	3	4	9
k. dealing with your health insurance	1	2	3	4	9
l. your citizen or immigration status	1	2	3	4	9
m. your distrust of the health care system	1	2	3	4	9
n. having the time needed to have the tests and treatments	1	2	3	4	9

C7. These next questions are about your overall impressions of the care you have received at {HEALTH CLINIC}. I am going to read a list of statements. For each statement, please tell me whether you Strongly Agree, Agree, Disagree or Strongly Disagree.

	Strongly Agree	Agree	Disagree	Strongly Disagree	NA
a. I would recommend this {CLINIC} to a friend who has similar needs.	1	2	3	4	9
b. The location of this {CLINIC}is convenient.	1	2	3	4	9
c. Overall, I am satisfied with this {CLINIC}.	1	2	3	4	9
d. In general, I am doing better because of this {CLINIC}.	1	2	3	4	9

Dintro. We would like to learn about the type of health care you get from {HEALTH CARE CLINIC}. Your answers will be kept confidential and will not be shared with your physician or clinic.

Over the past year, when I received health care, I was:

D1. Told about things I should do to improve my health.

1 ALL THE TIME
2 MOST OF THE TIME
3 SOME OF THE TIME
4 NONE OF THE TIME
9 NA

D2. Satisfied that my care was well organized.

1 ALL THE TIME
2 MOST OF THE TIME
3 SOME OF THE TIME
4 NONE OF THE TIME
9 NA

D3. Showed what I could do to take care of myself.

1 ALL THE TIME
2 MOST OF THE TIME
3 SOME OF THE TIME
4 NONE OF THE TIME
9 NA

D4. Helped to set specific goals to improve my eating, exercise or other health habits.

1 ALL THE TIME
2 MOST OF THE TIME
3 SOME OF THE TIME
4 NONE OF THE TIME
9 NA

D5. Asked questions about my health habits.

1 ALL THE TIME
2 MOST OF THE TIME
3 SOME OF THE TIME
4 NONE OF THE TIME
9 NA

D6. Helped to plan ahead so I could take care of my health in the future.

1 ALL THE TIME
2 MOST OF THE TIME
3 SOME OF THE TIME
4 NONE OF THE TIME
9 NA

D7. Contacted after a visit to see how things were going.

1 ALL THE TIME
2 MOST OF THE TIME
3 SOME OF THE TIME
4 NONE OF THE TIME
9 NA

D8. Encouraged to attend programs in the community that could help me.

1 ALL THE TIME
2 MOST OF THE TIME
3 SOME OF THE TIME
4 NONE OF THE TIME
9 NA

These next few questions are about your experiences at the {HEALTH CLINIC}.

E1. In the past year, how often did you feel the care you received was well coordinated?

1 ALL THE TIME

2 MOST OF THE TIME

3 SOME OF THE TIME

4 NONE OF THE TIME

9 NA

E2. How often were you confused about roles of different service providers?

1 ALL THE TIME

2 MOST OF THE TIME

3 SOME OF THE TIME

4 NONE OF THE TIME

9 NA

E3. How often did your main health care provider seem to be communicating with your other providers?

1 ALL THE TIME

2 MOST OF THE TIME

3 SOME OF THE TIME

4 NONE OF THE TIME

9 NA

We are almost done. I have a few final questions about you.

F1. In what year were you born?

1900-1993> ENTER YEAR

9999 NA

F2. What is the highest year of education you completed?

1	8TH GRADE OR LESS
2	SOME HIGH SCHOOL, BUT DID NOT GRADUATE
3	HIGH SCHOOL GRADUATE OR GED
4	SOME COLLEGE OR 2-YEAR DEGREE
5	4-YEAR COLLEGE GRADUATE
6	MORE THAN 4-YEAR COLLEGE DEGREE
9	NA

F3. Are you currently married, separated, divorced, widowed or have you never been married?

Married	[ ]
Separated	[ ]
Divorced	[ ]
Widowed	[ ]
Never Married	[ ]

{If F3 = "Married", do not ask F3ba, F3bb, F3bc}
{If F3 ="Never Married", do not ask F3bb}
F3b. Please answer yes or no to each of the following questions. Do you currently live with…

	YES	NO
a. no one else, that is, you live alone?	[ ]	[ ] {If YES, Skip to F4}
b. a husband or wife?	[ ]	[ ]
c. a partner?	[ ]	[ ]
d. other adult relatives?	[ ]	[ ]
e. your children?	[ ]	[ ]
f. children that are not yours?	[ ]	[ ]
g. friends or roommates?	[ ]	[ ]
h. someone else?	[ ]	[ ]

F4. Are you currently working at a full-time job, a part time job, or not working at all?

Full-time job	[ ] {Skip to F7}
Part-time job	[ ] {Skip to F7}
Not working	[ ]

F5. Are you currently looking for work?

1 YES

2 NO

F6. Are you currently retired?

1 YES

2 NO

F7. Are you currently a full-time student, a part-time student or not a student at all?

Full Time Student	[ ]
Part Time Student	[ ]
Not a Student	[ ]

F8. In the next two months, we will invite 30 participants in this phone survey to be interviewed in person about their experiences with the services at a HEALTH CENTER. The questions in the in-person interviews will be somewhat more in depth than those in this phone survey. All responses will be confidential. People will be selected at random for these in-person interviews from phone survey participants at several health centers. The interviews will be conducted by trained assistants from the University of Massachusetts at a time and place of your choosing and can be in either English or Spanish. They will take about 30 minutes. You will not be called and asked to participate in an in-person interview unless you agree now that a research assistant can call you about an interview.

F9. Do you agree to be called to be invited to participate in an in-person interview in the next two months?

1 YES

5 NO

9 NA

IF YES Is this the best number to reach you? ____________(alternative #)

F10. THIS INTERVIEW WAS CONDUCTED IN

1 ENGLISH

2 SPANISH

3 PORTUGESE

Thank you for your time and cooperation.
